# Health-related quality of life in children after surgical repair of esophageal atresia: a cross-sectional study in China

**DOI:** 10.3389/fped.2023.1332979

**Published:** 2024-01-09

**Authors:** Zhong Feng, Haitao Zhu, Weijing He, Xueni Peng, Runnan Gao, Yuxin Tian, Xuqing Cao, Gong Chen, Kuiran Dong, Shan Zheng, Chun Shen

**Affiliations:** Department of Pediatric Surgery, Children’s Hospital of Fudan University, Shanghai, China

**Keywords:** esophageal atresia, quality of life, questionnaire, PedsQL^™^ 4.0, EA-QOL questionnaire

## Abstract

**Objective:**

To investigate health-related quality of life (HRQOL) in patients after surgical repair for esophageal atresia (EA) and identify its potential influencing factors.

**Methods:**

A total of 102 EA children who had previously visited our hospital participated in this cross-sectional study. Basic data and disease data of the patients were collected. The HRQOL was measured with the Pediatric Quality of Life Inventory^™^4.0 (PedsQL^™^4.0) and EA-QOL questionnaire and ranked on a reverse 0–100 scale, with a higher number indicative of a better HRQOL perception. The scores of PedsQL^™^4.0 in children with EA were collected and compared with that of the demographically matched healthy control group. Meanwhile, the condition-specific HRQOL of EA was analyzed by the EA-QOL questionnaire, and the potential clinical factors that influenced the HRQOL were determined by the generalized linear model.

**Results:**

The group of EA and control reached a similar score in the generic PedsQL^™^4.0 (EA group: 86.55 ± 9.69; control group: 89.41 ± 6.54; *p* = 0.670). There was no significant difference between the EA group and the control group in other domains except the school functioning. Condition-specific HRQOL in the 2–7-year-old group had the highest score in social isolation and stress domain and the lowest score in the physical health and treatment domain, with an overall quality of life score of 83.48 ± 10.22. The scores of the 8–17-year-old group were relatively high in social relationships and health and well-being and lowest in the eating domain, with an overall quality of life score of 89.43 ± 8.57. Heart malformation, complicated esophageal surgery history, respiratory symptoms,and digestive symptoms in the past 1 month were the main factors affecting the HRQOL of children aged 2–7 years. Complicated esophageal surgery history, respiratory symptoms, and digestive symptoms in the past 1 month were the main factors affecting the HRQOL of children aged 8–17 years.

**Conclusions:**

The findings suggest that patients with EA generally had a good HRQOL. However, EA children with postoperative complications and associated symptoms have lower scores in the EA-QOL questionnaire.

## Introduction

Esophageal atresia (EA) is one of the most serious congenital gastrointestinal developmental malformations, requiring neonatal surgery, with an incidence rate of 2.4:100,000 births (1). With the continuous improvement in surgery and neonatal care, the postoperative survival rate of EA children has exceeded 90% (2). However, postoperative EA patients are at risk of gastrointestinal and respiratory complications, which affects the long-term prognosis of some children (3). The health-related quality of life (HRQOL) after surgical repair has become the focus of follow-up and research.

HRQOL refers to a patient's subjective experience of daily life their physical, mental, and social functioning under the influence of the disease itself, and with medical interventions. These HRQOL instruments are generic or condition-specific (4). The generic instruments have wider applicability, which enable applications to compare differences between patients and healthy people (5). In the past, due to the lack of condition-specific instruments, HRQOL in EA patients after surgical repair was assessed using generic instruments, but these scales may ignore the influence of heterogeneity and the concomitant factors of EA. A few years ago, an EA-specific HRQOL instrument named EA-QOL questionnaire was developed in Sweden and Germany, with confirmed feasibility, reliability, and validity (6). It is reasonable to hypothesize that clinical factors associated with birth characteristics, surgery, and EA-related digestive and respiratory morbidity would affect these HRQOL domains negatively. However, only a few studies in the literature use this instrument to evaluate the postoperative quality of life in children with EA (7, 8). Due to the heterogeneity of EA, larger cohorts need to be investigated to ensure data reliability.

To fill this gap in our knowledge and yield more information for future studies, we aimed to evaluate generic as well as condition-specific HRQOL in Chinese children with EA. Moreover, we explored the risk factors that affect the HRQOL of children with EA to provide more evidence to improve the HRQOL.

## Methods

### Study design and participants

The cross-sectional survey was conducted during the period from September to December in 2022 and approved by the Ethics Committee of the Children's Hospital of Fudan University. All procedures were in compliance with the principles of the Declaration of Helsinki (as revised in 2013) and followed approved research protocols. The families of children who were accepted for the initial surgery in our hospital were selected as the study group. The medical records of the children were complete and their current ages ranged from 2 to 18. Meanwhile, we recruited children who were enrolled in the outpatient department for postoperative follow-up of oblique inguinal hernia as a healthy control group based on the demographic characteristics of the EA group. These healthy control group members did not have other disease or other abnormalities. All children in our study had been taken care of in the same way as in all other centers in the world. The cross-sectional survey was completed through the outpatient service, telephone, or internet. All the parents or guardians of the participating children read and signed the informed consent prior to participation. All surveys and questionnaires were completed by proxy-report.

### Instruments

#### General information collection

A parent reported that the general information questionnaire developed by our team was used to obtain information on sociodemographic factors and health-related data of the children and their families. All questions were answered using a 1-month recall period, mainly including a child's gender, age, sleep quality, acute or chronic disease symptoms, as well as the age, gender, address, education level, and health status of the adult caregiver or parent. For children with EA, clinical data including birth weight, gestational week, combined deformity, therapies, postoperative complication, and the child's digestive and respiratory symptoms during the previous 1 month were collected.

#### PedsQL™ 4.0 generic core scale

The Pediatric Quality of Life Inventory™4.0 (PedsQL™ 4.0) was originally developed in the English language by Varni et al. (9, 10) and has a reliable and validated Chinese version. The PedsQL™ 4.0 generic core scale is adapted to children and adolescents aged 2–18 years and consists of questions related to four functional domains, including physical, emotional, social, and school functioning, with a total of 23 items. Each item is a question on the frequency of something that happend in the previous month, using a five-point Likert scale for responses. All items are linearly transformed to the 0–100 scale, with higher scores indicating better HRQOL. The normal values of PedsQL 4.0 were derived from previously conducted validation studies and a score below 80 could be considered as impaired quality of life (11–13).

#### EA-QOL questionnaire

The condition-specific EA-QOL questionnaire was originally developed for the Swedish-German study by Dellenmark-Blom et al. (14) and already has a standardized and content-validated Chinese Mandarin version (15). Our team also confirmed that the Chinese version of the scale showed good reliability and validity in the preliminary study. The EA-QOL questionnaire have two versions: (1) one for 2–7-year-old children and (2) another for 8–17-year-old children. The 2–7-year-old version includes three domains: eating, physical health and treatment, and social isolation and stress, for a total of 17 items; and the 8–17 year-old version includes four domains: eating, social relationships, body perception, and health and well-being, for a total of 22 items. Each item is a question on the frequency of something that happened in the previous month, according to a five-point Likert scale. All items are linearly transformed to the 0–100 scale. Higher scores indicate better HRQOL. Based on experience from previous studies, a score below 80 could be considered as impaired quality of life (16).

### Statistical analysis

The general characteristics of the patients with EA are presented using descriptive statistics. The missing data are treated by the multiple imputation methods. For quantitative data with a normal distribution, the mean ± standard deviation (SD) is presented. Independent sample *t*-tests were used for comparisons of differences between groups. For quantitative data with a non-normal distribution, medians with interquartile intervals are presented. Mann–Whitney *U* tests were used for comparisons of differences between groups. For categorical data, comparisons between groups were performed using chi-square (*χ*^2^) tests or Fisher's precision probability test. The generalized linear model was then used to identify independent determinants of the HRQOL. A value of *P* < 0.05 was considered to be statistically significant. All statistical analyses were performed with the SPSS 28.0 statistical software (SPSS Inc., Chicago, USA).

## Results

### Children’s characteristics and questionnaire response

In total, 207 questionnaires were effectively collected including for 102 children with EA (46 male, 56 female) and 105 healthy children (48 male, 57 female). The mean age of the patients was 7.17 ± 3.30 years, median age was 7 years, range from 2 months to 14 years (IQR 4–10). The mean age of the healthy children was 7.18 ± 3.25 months, median age was 7 years, range from 2 months to 14 years (IQR 4–10). Table 1 shows the baseline characteristics of the children with EA and those in the control group. Age, gender, and family factors such as education level of the parents were not significantly different between the EA group and the control group.

**Table 1:** Baseline characteristics of the children in the EA and control groups.

	EA group	Control group	*P* value
	*n* = 102	*n* = 105	
Age, year	7.17 ± 3.30	7.18 ± 3.25	0.798
Gender			0.432
Male	46	48	
Female	56	57	
Place of residence			0.952
Urban area	53	55	
Rural area	49	50	
Relationship between respondents			0.259
Father	25	19	
Mother	77	86	
Education level of the respondent			0.321
Elementary education	15	14	
Secondary education	45	37	
Higher education	42	54	

The demographic details and clinical data of the EA group are provided in Table 2. Among them, 79(77.5%) patients were full-term at birth and 80(78.4%) patients had normal birth weight. Furthermore, 29(28.4%) patients had accompanied heart malformation and 15(14.7%) children had other associated abnormalities. In addition, 57(55.9%) patients had thoracoscopic surgery, and 15(14.7%) patients had a history of complex esophageal surgery, including eight cases of long gap EA (seven patients received delayed primary anastomosis and one received colon replacement), two cases of esophageal re-anastomosis due to refractory stricture, and five cases of recurrent tracheoesophageal fistulas. A total of 62(60.8%) children had gastrointestinal symptoms at ordinary times, primarily mild dysphagia, which manifested as slow eating (eating speed lagging behind the healthy children in the same age and with each meal lasting for more than half an hour). A total of 36 (35.2%) cases had symptoms of reflux or heartburn after eating. Most of them only had slight symptoms, which could be controlled by pillow padding. Two patients had severe reflux symptoms and had to further undergo fundus folding surgery after being confirmed by esophagography as having gastroesophageal reflux. Their reflux symptoms are much improved currently. In addition, 32(31.3%) children had respiratory symptoms, including coughing, chest distress, or polypnea when resting or active, or frequent respiratory infections.

**Table 2:** Demographic details and clinical data of the EA group (*n* = 102).

	*n* (%)/*M*
Male	46(45.1)
Age, year	7.17 ± 3.30
Gestational age ≥37 weeks	79(77.5)
Birth weight ≥2.5 kg	80(78.4)
Thoracoscopic surgery	57(55.9)
Complex esophageal surgery	15(14.7)
Esophageal dilatation ≥3 times	42(41.2)
Cardiac malformation	29(28.4)
Other deformity	23(22.5)
Digestive symptoms	62(60.8)
Respiratory symptoms	32(31.3)

### Comparison of PedsQL^™^4.0 scores between children with EA and control group

The results showed that children with EA had the highest score in the physical functioning domain and the lowest score in the school functioning domain, as shown in Table 3. They had a good quality of life in the areas of physiological, emotional, and social functioning, and there was no significant statistical difference compared with the control group. However, the scores in the areas of school functioning were still significantly lower than that of the control group, although the average score exceeded 80 points (*P* < 0.05). Compared with the control group, there was no significant difference in the overall score of EA children (*P* > 0.05).

**Table 3:** Score for each dimension of the PedsQL^TM^ 4.0 in the EA and control groups.

	EA group	Control group	*t*	*P* value
Physical functioning	90.39 ± 15.35	92.09 ± 13.47	−0.997	0.267
Emotional functioning	87.35 ± 10.59	88.29 ± 9.87	−0.656	0.200
Social functioning	87.84 ± 12.33	86.36 ± 11.44	0.896	0.172
School functioning	81.33 ± 13.97	91.2 ± 6.68	−6.508	0.001
Total scores of PedsQL^™^ 4.0	86.55 ± 9.69	89.41 ± 6.54	−0.187	0.670

### Scores of the EA-QOL questionnaire in children with EA

A total of 65 questionnaires were collected from the 2–7-year-old group. The results showed that the HRQOL scores were the lowest in the area of physical health and treatment, with a score of 80.30 ± 13.44, and 33 (52.31%) children had a score more than 80 points. The children had the highest average scores of 88.08 ± 12.24 in the areas of social isolation and stress domain, and 56 (86.15%) children had a score more than 80 points. The score in the area of eating was 82.07 ± 11.83, and 38 (58.46%) children had a score more than 80 points. The overall HRQOL score was 83.48 ± 10.22, and 36 (55.38%) cases scored above 80 points.

A total of 37 questionnaires were collected from the 8–17-year-old group. The results showed that the scores of the 8–17-year-old children with EA in the domain of eating were relatively low, 82.91 ± 13.62, and 24 (64.8%) children were above 80 points. The scores were relatively high in the domain of social relationships and health and well-being. The scores of the two domains were 91.04 ± 9.04 and 92.83 ± 10.38, 35 (94.59%) and 34 (91.89%) cases were above 80 points. In the domain of body perception, the score was 84.43 ± 11.69, with 34 (91.89%) cases higher than 80 points. The overall HRQOL score was 89.43 ± 8.57, and 32 (86.49%) cases were above 80 points. The scores of each domain are shown in Table 4.

**Table 4:** Scores of EA-QOL questionnaire in children with EA.

Domain	Scores	Scores > 80, *n* (%)
Children 2–7 years old group (*n* = 65)		
Eating	82.07 ± 11.83	38(58.46)
Physical health and treatment	80.30 ± 13.44	33(52.31)
Social isolation and stress	88.08 ± 12.24	56(86.15)
Total score	83.48 ± 10.22	36(55.38)
Children 8–17 years old group (*n* = 37)		
Eating	82.91 ± 13.62	21(56.76)
Social relationships	91.04 ± 9.04	35(94.59)
Body perception	84.43 ± 11.69	27(72.97)
Health and well-being	92.83 ± 10.38	34(91.89)
Total score	89.43 ± 8.57	32(86.49)

### Factors influencing the quality of life of patients with EA

The generalized linear model was used to further explore influencing factors of the 2–7 and 8–17-year-old groups of children, respectively. As shown in Tables 5, 6, heart malformation, complicated esophageal surgery history, respiratory symptoms, and digestive symptoms in the past 1 month were the main factors affecting the quality of life of children aged 2–7 years. Complicated esophageal surgery history, respiratory symptoms, and digestive symptoms in the past 1 month were the main factors affecting the quality of life of children aged 8–17 years. Besides, the relationship between thoracoscopic surgery and body perception is presented in Supplementary Table S1.

**Table 5:** Analysis of single factors influencing HRQOL of patients with EA.

Variables	*n*	Yes	*n*	No	*P* value
		Mean ± SD		Mean ± SD	
Children 2–7 years old group (*n* = 65)					
Female	37	83.81 ± 10.26	28	83.04 ± 10.53	0.920
Other deformity	14	80.51 ± 9.04	51	84.30 ± 10.57	0.259
Cardiac malformation	18	78.12 ± 7.93	47	85.53 ± 10.44	0.023
Thoracoscopic surgery	43	82.80 ± 9.99	22	84.81 ± 10.52	0.079
Complex esophageal surgery	7	71.55 ± 3.96	58	84.92 ± 9.90	0.034
Digestive symptoms	42	76.95 ± 5.97	23	95.05 ± 3.56	0.001
Respiratory symptoms	21	75.76 ± 5.04	44	87.16 ± 10.16	0.035
Children 8–17 years old group (*n* = 37)					
Female	19	89.96 ± 8.59	18	88.88 ± 7.76	0.851
Other deformity	9	87.12 ± 6.86	28	90.18 ± 9.04	0.940
Cardiac malformation	11	87.80 ± 8.20	26	90.13 ± 8.79	0.530
Thoracoscopic surgery	14	89.84 ± 8.52	23	89.19 ± 8.78	0.122
Complex esophageal surgery	8	77.06 ± 3.76	29	92.85 ± 5.93	0.001
Digestive symptoms	20	83.32 ± 6.76	17	98.63 ± 3.08	0.001
Respiratory symptoms	11	79.38 ± 5.39	26	93.69 ± 5.56	0.044

**Table 6:** The independent risk factors of impaired condition-specific HRQOL in children with EA.

	Estimate	Std. error	*t* value	Pr(>|*t*|)
Children 2–7 years old group				
Female	0.0418	1.6900	0.0250	0.9804
Other deformity	0.8308	2.5024	0.3320	0.7411
Cardiac malformation	−0.1468	0.0646	−2.2736	2.66×10^−2^
Thoracoscopic surgery	2.6747	2.3082	1.1590	0.2515
Complex esophageal surgery	−0.1762	0.0821	−2.1465	3.60×10^−2^
Digestive symptoms	−0.3095	0.0604	−5.1219	3.49×10^−6^
Respiratory symptoms	−0.1261	0.0610	−2.0665	4.32×10^−2^
Age	−0.1184	0.5187	−0.2280	0.8202
Children 8–17 years old group				
Female	0.7505	1.3091	0.5730	0.5710
Other deformity	−0.3264	1.5825	−0.2060	0.8381
Cardiac malformation	0.8407	1.4407	0.5840	0.5642
Thoracoscopic surgery	1.9280	1.3095	1.4720	0.1521
Complex esophageal surgery	−0.2955	0.0667	−4.4303	1.03×10^−4^
Digestive symptoms	−0.2677	0.0507	−5.2840	8.70×10^−6^
Respiratory symptoms	−0.1434	0.0653	−2.1959	3.55×10^−2^
Age	0.1649	0.3996	0.4130	0.6829

## Discussion

Despite advances in the prognosis of EA, knowledge on patients’ HRQOL is relatively sparse in the literature. This study reported postoperative quality of life in children with EA in the Chinese population, which fills several knowledge gaps of the medium and long-term prognosis in China. First, these data indicated children with EA had a good quality of life, generally no different from normal children. Second, the study showed the the HRQOL of children with EA aged 2–7 years was mainly impaired in the Physical health and Treatment domain, while those aged 8–17 years were in the eating domain. Finally, a history of complex esophageal surgery, and postoperative digestive and respiratory symptoms, were identified as major factors affecting the condition-specific HRQOL of EA patients throughout childhood, while cardiac malformation primarily affected children aged 2–7 years. Our study used a combination of generic and specific instruments to evaluate the HRQOL of children with EA, providing more comprehensive information for such studies.

Our study adopted the most commonly used generic scale for children, the PedsQL™ 4.0, to evaluate the HRQOL of EA survivors. Previously, some centers have conducted studies on generic HRQOL, with conflicting results. Legrand et al. (17) described that overall HRQOL in children with EA was reduced as compared with healthy children, with impairments in all the four domains of PedsQL™ 4.0. Children with dyspnea and dysphagia had impaired emotional functioning, and children with combined malformations, especially heart disease, had lower school performance scores. Another cohort was consistent and proposed that children with EA had significantly impaired emotional and social functioning. The average score of emotional functioning in all age groups was 52.73–60.05, which was the lowest in all domains (18). In addition, some researchers have reported different degrees of impaired HRQOL in different areas (19, 20). However, a study reported that overall HRQOL among children with EA was similar to that of a healthy reference group (21). Our research showed that children with EA had a good quality of life in the physiological, emotional, and social functioning domains, and there were no significant statistical differences compared with the control group, except for significantly lower scores in the school functioning domain. The overall quality of life score of children with EA was not significantly different from that of the control group. The HRQOL condition in our study appeared to be better than in some of the aforementioned literature reports (16, 17). In this study, the cases of EA combined with severe malformations, chromosome abnormalities, and severe disease status were more likely to be abandoned or lost to follow-up, while cases with fewer malformations, no chromosome abnormalities, and mild disease status were more likely to adhere to treatment and obtain long-term follow-up, which may lead to better prognosis of follow-up and evaluation in this study.

Our study also provides a preliminary reference for EA-specific HRQOL in the Chinese population. In previous reports from other countries, four of six articles stated that the 2–7-year-old group had the lowest scores in the physical health and treatment domain (6, 22–24), one article described lowest scores in the social isolation and stress domain (25), and one article did not report the specific HRQOL of the 2–7-year-old group (26). All six articles reported that the 8–17-year-old group had the lowest scores in the eating domain (6, 22–26). Our results were similar to these studies. In the 2–7-year-old group, young EA postoperative patients still need to be hospitalized repeatedly owing to esophageal anastomotic stenosis, combined with heart defects, tracheomalacia, and recurrent respiratory symptoms, which could lead to an impaired physical health and treatment domain. In the 8–17-year-old group, EA postoperative patients in this age group have received treatment and shown improvement for problems such as anastomotic stenosis, combined deformity, tracheomalacia, and recurrent respiratory symptoms that they faced at a younger age, and have entered a stable stage, while the problem of postoperative esophageal motor dysfunction has become increasingly prominent.

Our study also identified several potential factors associated with lower condition-specific HRQOL, including a history of complex esophageal surgery, digestive and respiratory symptoms in both groups, and cardiac malformation in the 2–7-year-old group. Although prior studies have identified several acknowledged factors associated with lower generic HRQOL, including prematurity, respiratory and digestive symptoms, gastrostomy procedure, and severe EA (17, 20, 21, 27), only a few studies in the literature have described potential factors affecting condition-specific HRQOL (16, 28). One report suggested that the condition-specific HRQOL domain of pediatric patients after EA repair was negatively affected by digestive symptoms throughout childhood, while respiratory symptoms primarily affected children aged 2–7 years (16). In addition, no primary anastomosis, esophageal dilatation, as well as gastrostomy insertion secondary to long-gap EA or severe gastroesophageal reflux predicted worse scores related to eating domain in all ages; associated anomalies were related to worse body perceptions (16). Another research stated that days of discharge after EA repair and concomitant abnormalities were significantly negatively correlated with HRQOL scores in the 2–7 age group, while relevant respiratory and digestive tract symptoms such as airway infection, swallowing difficulties, and heartburn were associated with lower HRQOL scores in the 8–17 years group (28). Our results support the relevant opinions of the previous literature. Besides, we found thoracoscopic surgery was related to better scores in the body perception domain, which has rarely been discussed in the previous HRQOL studies. Only one study mentioned that there are no significant differences in the HRQOL of children after school age between the traditional and thoracoscopic surgery groups (29). Our findings support that thoracoscopic repair has the advantages of less trauma and superior cosmetic results compared with the thoracotomy (30), which could improve body perception.

Our study also had some limitations. First, in accordance with the wishes of the majority of the respondents, we adopted parent-proxy questionnaire in this investigation. This could create potential bias, although some studies have shown a good parent–child agreement (26). Second, as a single-center pilot study conducted on Chinese patients, the generalizability of the result and value in practical applications needs to be further verified. Third, the type of EA and the surgical options (early vs. delayed primary anastomosis) may also impact the HRQOL. Because of the limited sample size, these factors have not been fully explored in this study. In the future, we hope to continue this study by enlarging the sample size and seeking the cooperation of other centers to further verify the accuracy of these results.

In conclusion, patients with EA generally have a good quality of life after surgery, although children with postoperative complications and associated symptoms have lower scores in the EA-QOL questionnaire. Early repeated hospitalizations and surgeries may have long-term effects on the physical health. Children with EA with symptoms and scars have more negative perceptions of their bodies. At the same time, complex esophageal surgeries such as fundoplication worsen esophageal outlet obstruction and esophageal functional disorders, negatively affecting the eating domain of patients’ quality of life. Therefore, postoperative follow-up of patients with EA is necessary, early attention should be paid, and their quality of life should be improved through psychological counseling, health education, and by alleviating complications.

## Data Availability

The original contributions presented in the study are included in the article/Supplementary Material, further inquiries can be directed to the corresponding author.
