# Supplementary material for: Health-related quality of life in children after surgical repair of esophageal atresia: a cross-sectional study in China
Source: Front Pediatr. 2024 Jan 9;11:1332979. doi: 10.3389/fped.2023.1332979 (PMC10803562; doi:10.3389/fped.2023.1332979)
Supplement: Supplementary file 1 [file Table1.docx]

| **Table S1. The independent risk factors of impaired body perception field in children with EA (aged 8-17).** | | | | |
| --- | --- | --- | --- | --- |
|  | Estimate | Std. Error | t value | Pr(>\|t\|) |
| thoracoscopic surgery | 0.1624 | 0.0617 | 2.6326 | 1.28E-02 |
| complex esophageal surgery | -0.2271 | 0.0830 | -2.7371 | 9.91E-03 |
| digestive symptoms | -0.3321 | 0.0680 | -4.8832 | 2.60E-05 |
